# Impact of BMI on fertility in an otherwise healthy population: a systematic review and meta-analysis

**DOI:** 10.1136/bmjopen-2023-082123

**Published:** 2024-11-01

**Authors:** Florence Turner, Simon G Powell, Hannan Al-Lamee, Anjali Gadhvi, Ellen Palmer, Andrew Drakeley, Victoria S Sprung, Dharani Hapangama, Nicola Tempest

**Affiliations:** 1 Centre for Women's Health research, Department of Women's and Children's Health, Institute of Life Course and medical Sciences, University of Liverpool, Member of Liverpool Health Partners, Liverpool, L8 7SS, UK; 2 Liverpool University Hospitals NHS Foundation Trust, Liverpool, L7 8XP, UK; 3 Liverpool Women's NHS Foundation Trust, Member of Liverpool Health Partners, Liverpool, L8 7SS, UK; 4 The Hewitt Fertility Centre, Liverpool Women's Hospital, Liverpool, L8 7SS, UK; 5 Research Institute for Sport & Exercise Sciences, Liverpool John Moores University, Liverpool, L3 3AF, UK

**Keywords:** Body Mass Index, Subfertility, Reproductive medicine

## Abstract

**Abstract:**

**Background:**

An increased body mass index (BMI) can lead to subfertility; however, current literature fails to exclude the effect of other confounding medical conditions, raising questions regarding the direct link between increased BMI and fertility outcomes.

**Objectives:**

To conduct a systematic review and meta-analysis to elucidate the effects of increased BMI on fertility outcomes in females with no other comorbidities.

**Search strategy:**

A comprehensive search was conducted using EMBASE, MEDLINE and the Cochrane library from January 2000 until July 2023.

**Data collection and analysis:**

Two authors independently conducted data extraction and assessed study quality. Odds ratio (OR) (dichotomous data), standardised mean difference (SMD) (continuous data) and 95% CIs were calculated.

**Main results:**

Nine eligible studies were identified: one natural conception and eight assisted reproductive technology (ART). Aggregated data revealed women with BMI ≥25 were less likely to attain clinical pregnancy (OR 0.76, 95% CIs 0.62 to 0.93, p=0.007), with BMI ≥30 associated with a further decreased likelihood of clinical pregnancy (OR 0.61, 95% CIs 0.39 to 0.98, p=0.04). Women with raised BMI required longer duration of stimulation (SMD=0.08, 95% CIs 0.00 to 0.16, p=0.04) and obtained reduced oocytes (SMD=−0.11, 95% CIs −0.18 to −0.04, p=0.002).

**Conclusions:**

These data demonstrate an adverse impact of being overweight/obese on ART outcomes in women with no other diagnosed medical comorbidities and highlight the distinct lack of data concerning the effects of isolated obesity on natural conception. Infertility represents an enormous burden for couples and society; it is essential to identify and tackle modifiable risk factors to improve chances of conception.

**PROSPERO registration number:**

CRD42022293631.

**STRENGTHS AND LIMITATIONS OF THIS STUDY**

- Strict eligibility criteria removed the influence of endocrine, metabolic or gynaecological disorders that have their own direct effects on body mass index (BMI)/fertility.
- Only one paper investigated the effects of raised BMI on natural conception; therefore, conclusions could only be drawn for assisted reproductive technology outcomes.
- Results are based on data from studies of varying quality and risk of bias.
- A small number of studies reported on live birth rate (n=3) with outcome reporting in reproductive medicine studies an ongoing area of debate.

## Introduction

 Infertility is defined as the failure to achieve a pregnancy after 12 months or more of regular unprotected sexual intercourse and is a disease of the male and or female reproductive system.^1^

Obesity is a growing problem globally. In 2016, more than 1.9 billion adults were overweight (body mass index (BMI) 25.0–29.9 kg/m^2^), and over 650 million were obese (BMI ≥30.0 kg/m^2^).^2^ The proportion of adults defined as overweight or obese is expected to rise significantly in the coming years,^3^ and by 2050, in the UK, costs to the National Health Service (NHS) attributable to being overweight and obese have been estimated to reach £9.7 billion a year.^4^

Several systematic reviews have been conducted to assess the impact of being overweight/obese on assisted reproduction,^5^,^7^ but not on natural fecundability. A high BMI has been shown to negatively affect live birth rates (LBR), miscarriages and ovarian stimulation; however, conflicting evidence remains for whether obesity negatively affects implantation or clinical pregnancy rate (CPR).^7 8^ Noticeably, previous reviews have included studies in which females with medical comorbidities (such as polycystic ovaries and thyroid disease) were not excluded leaving it difficult to counsel women who are overweight/obese with no other comorbidities regarding their fertility.

With increasing rates of obesity recorded and the NHS currently spending approximately £68 million per year on in vitro fertilisation (IVF) treatments,^9^ it is necessary to explore and address modifiable fertility risk factors, where possible, and fully understand the effects of excess weight on fertility. The aim of this study was therefore to consolidate published data on the association between increased BMI and fertility in patients with no other diagnosed medical comorbidities.

## Methods

This review was registered with the International Prospective Register of Systematic Reviews under the registration protocol number CRD42022293631 and was reported according to the Preferred Reporting Items for Systematic Reviews and Meta-Analyses (PRISMA) and Meta-analysis of Observational Studies in Epidemiology guidelines.^10^

### Search strategy

A systematic search was performed using EMBASE, MEDLINE and the Cochrane CENTRAL Library. As significant advances have been made in managing females with subfertility, it would not be possible to compare management directly before and after the year 2000. Hence, studies published between 1 January 2000 and 1 July 2023 were included in this systematic review. The search string utilised a combination of exploded MeSH terms (Female OR Woman OR Women) AND (BMI OR Body mass index OR Overweight OR Obesity) AND (Infertility OR Conception OR Ovulation OR Anovulation Or Fecundity OR Assisted Reproduction OR Assisted Reproductive Technologies OR ART OR IVF OR ICSI) (online supplemental table 1). Results were filtered to English language studies only. Grey literature was not searched.

### Study selection and eligibility criteria

Results from the initial searches were collated, and duplicates were deleted. Title and abstract screening were completed independently by two authors (FT and AG) using the online software Rayyan,^11^ a semi-automated tool for initial screening in systematic reviews. Any disagreements were resolved by discussion and the involvement of a third senior author (NT). All original randomised and non-randomised studies assessing BMI in association with any pregnancy outcomes were included in this review. Studies were excluded if (1) they did not report on BMI or any associated fertility outcome, (2) they included any participants with comorbidities that may affect fertility such as PCOS and thyroid disease (we read all papers inclusion and exclusion criteria, and if women were included that had any comorbidities that may affect fertility we excluded the paper), (3) they were not written in the English language, (4) they were not full-text articles (including abstracts and incomplete datasets) and (5) they were not original research studies (including review articles, meta-analyses, case reports and conference abstracts). Full texts were retrieved, and reviews were completed independently by two authors (FT and AG). Each study was assessed for inclusion using the predetermined eligibility criteria. Any disagreements were resolved by discussion and the involvement of a third senior author (NT).

Additional studies were identified through forward and backward chaining of all studies included thus far. References to all relevant literature and systematic reviews identified by the initial search were also screened.

### Data extraction and synthesis

Data from included studies were extracted independently by two authors (FT and AG). Data included, but was not limited to, title, author, journal, year of publication, total number of participants, number of participants per BMI category, IVF/intra-cytoplasmic sperm injection (ICSI) cycle parameters (total gonadotrophin dose IU/L, duration of stimulation days, the mean number of mature oocytes retrieved and mean number of embryos transferred) and pregnancy outcomes (CPR, defined as the visualisation of a foetal heartbeat on ultrasound, miscarriage rate and LBR).

### Bias analysis

All studies included in the analysis were assessed for quality and risk of bias using the Newcastle-Ottawa scale,^12^ an assessment tool for risk of bias. Up to a maximum of nine points can be assigned for risk of bias over three domains: selection of study groups, comparability of groups and ascertainment of outcome.

### Statistical analysis

Review Manager V.5.4 (RevMan) software^13^ was used for statistical analyses. I^2^ was used to determine total variability, a fixed effects model was used where I^2^ was <50%, and a random effects model was used for I^2^ >50%. The Mantel–Haenszel method was applied for dichotomous data pooling, and the results were presented as an odds ratio (OR) with 95% CIs. For pooling of continuous data, the inverse variance method was used to generate a standardised mean difference (SMD) with 95% CIs. A p value <0.05 was deemed statistically significant.

### Patient and public involvement statement

No patients or public were involved with the design or conduct of this study, but we will disseminate the findings through our active women’s health Patient and Public Involvement group at the Liverpool Women’s Hospital and also across our social media.

## Results

### Eligible studies

**Figure 1:**
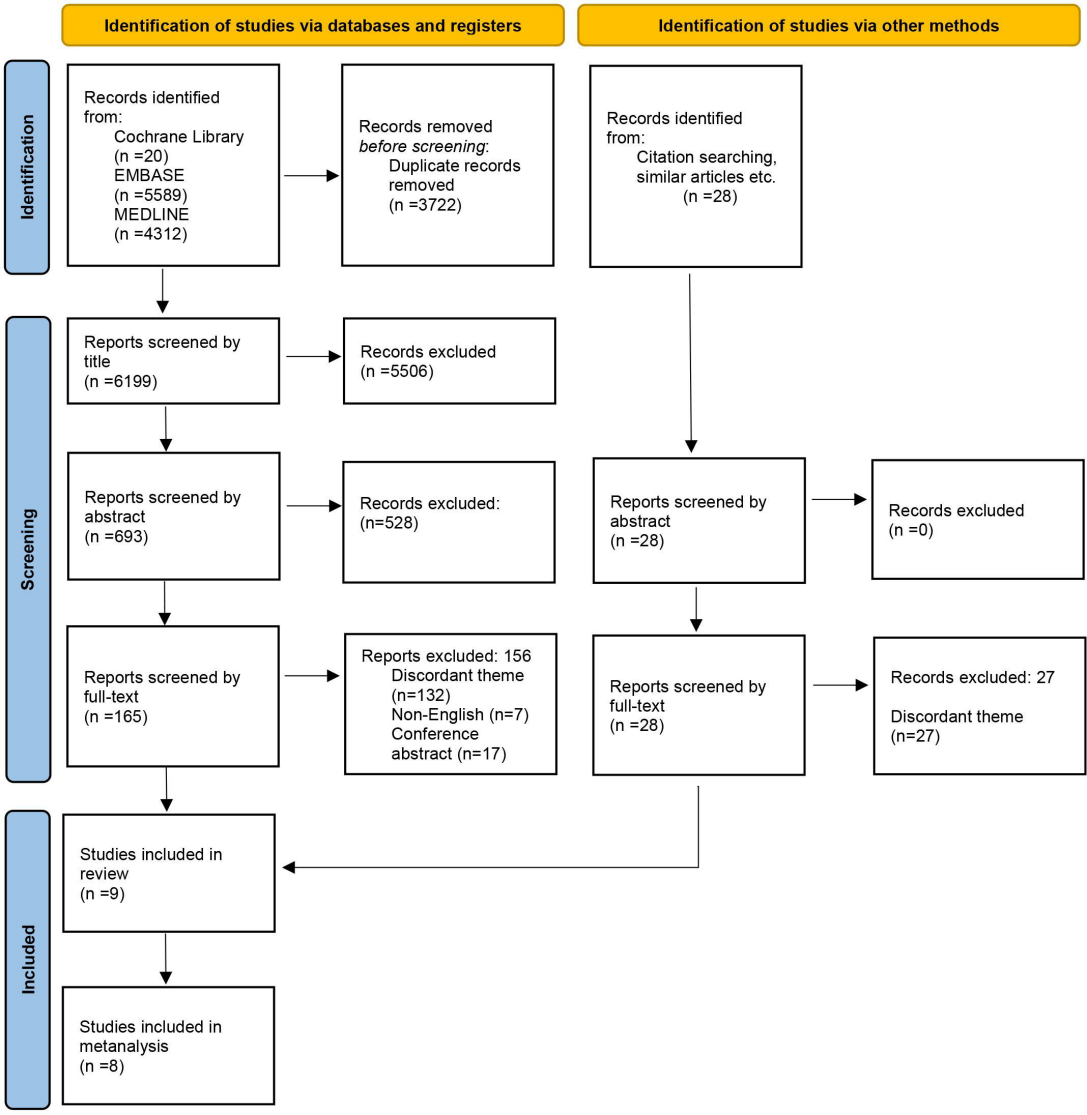
Preferred Reporting Items for Systematic Reviews and Meta-Analyses flow diagram illustrating systematic review search strategy. An initial of 7326 studies were filtered down to nine eligible studies using our strict inclusion criteria. One study was identified via forward and backward chaining. Eight studies were suitable for meta-analysis.

An initial database search identified 9921 studies, of which 3722 duplicates were identified and removed. Title screening identified 693 relevant studies, which were subsequently screened by abstract, leaving 165 studies remaining (figure 1). Following full-text screening, eight studies were identified to be eligible for inclusion. The references of these studies were screened, which identified one further eligible study, generating a total of nine studies for inclusion in the systematic review. Eight of the studies explored the effects of BMI on assisted reproductive technology (ART) outcomes, and only one paper^14^ identified the effects of BMI on natural conception, producing incomparable outcomes; hence, eight ART papers were included in the meta-analysis.

### Study characteristics

In total, 4108 cycles from 3770 patients undergoing ART were included. Eight studies were analysed; seven retrospective cohort studies and one prospective cohort study (online supplemental table 2). Studies used IVF and ICSI and were performed in China,^15 16^ Egypt,^17 18^ Turkey,^19 20^ Argentina^21^ and Brazil^22^ between 2000 and 2018. Six studies adhered to the standard WHO BMI categories^16^,^21^ (healthy, 18.5–24.9 kg/m^2^; overweight, 25.0 –29.9 kg/m^2^; obese, ≥30.0 kg/m^2^), while one study stratified patients using the Chinese standard^15^ (healthy, 18.5–23.9 kg/m^2^; overweight, 24.0–27.9 kg/m^2^; and obese, ≥ 28.0 kg/m^2^) and another using 19.0 kg/m^2^ as the minimum for a healthy BMI.^22^

### Risk of bias and quality assessment

Generally, there was a low risk of bias, with studies scoring six to eight out of a possible nine on the Newcastle-Ottawa Scale (online supplemental table 3). No obvious publication bias was identified by funnel plot analysis (online supplemental figure 1), but this should be interpreted with caution as only seven studies are plotted.

### Total gonadotrophin dosage

Seven studies^15^,^1719^ provided data for total gonadotrophin dose, four of which stated the specific gonadotrophin used, revealing a variety of gonadotrophins were utilised for ovarian stimulation (Gonal-F^15 19 22^ and Merional^17^).

Analysis of healthy versus overweight and healthy versus obese females showed no significant difference in total gonadotrophin dosage (SMD=0.04, 95% CIs −0.13 to 0.22, p=0.62) (online supplemental figure 2A) (SMD=0.33, 95% CIs −0.02 to 0.69, p=0.07) (online supplemental figure 2B), respectively. However, an overall trend of increasing total gonadotrophin dose with increasing BMI was observed in obese females (online supplemental figure 2B). All of those with a BMI ≥25.0 versus healthy BMI showed a trend toward requiring a larger total gonadotrophin dose (SMD=0.21, 95% CIs −0.09 to 0.51, p=0.17) (online supplemental figure 2C). Gonadotrophin dosage between studies displayed a high level of heterogeneity (I^2^ range: 76–94%) which was expected due to a variety of gonadotrophins used, with different methods of dosing.

### Duration of ovarian stimulation

Out of the eight eligible studies, six^15^,^1719^ provided data concerning the duration of ovarian stimulation in healthy versus overweight and healthy versus obese cohorts. One study^18^ provided data for healthy versus overweight and obese BMI combined. Comparison of overweight and healthy BMI showed similar duration of stimulation (SMD=0.03, 95% CIs −0.06 to 0.12, p*=*0.54) (online supplemental figure 3A), whereas those classified as obese were shown to require a longer duration of stimulation (SMD=0.20, 95% CIs 0.07 to 0.34, p=0.002) (online supplemental figure 3B). Overall, a BMI ≥25.0 was associated with requiring a longer duration of ovarian stimulation (SMD=0.08, 95% CIs 0.00 to 0.16, p=0.04) (online supplemental figure 3C).

### Number of mature oocytes retrieved

**Figure 2:**
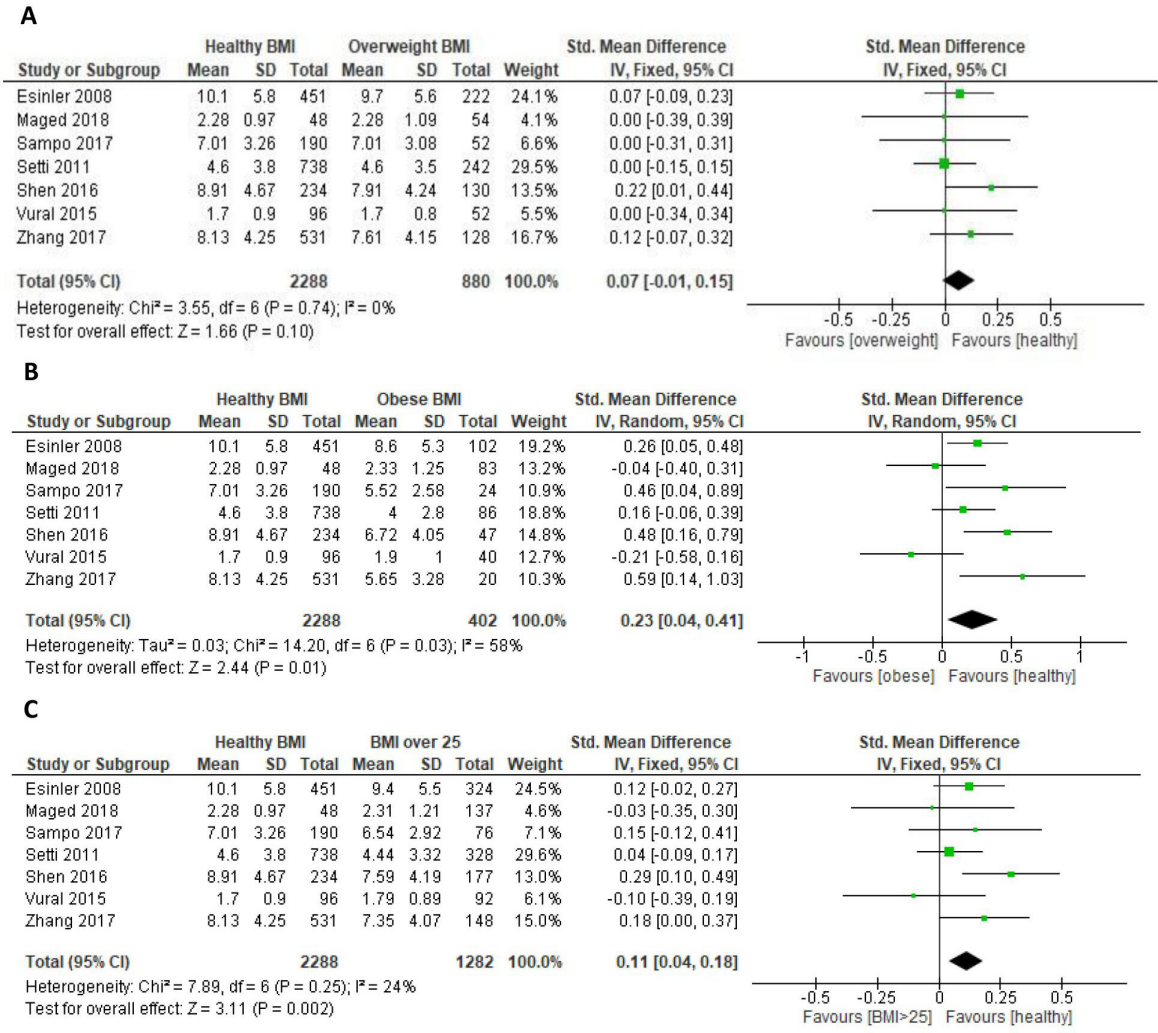
Analysis of the effect of body mass index (BMI) on the number of mature oocytes retrieved. (**A**) Healthy versus overweight BMI. (B) Healthy versus obese BMI. (C) Healthy versus BMI ≥25 kg/m^2^. df, degrees of freedom; Std., standard; IV, inverse variance.

Seven of the eight studies provided data for meta-analysis on the number of mature oocytes retrieved.^15^,^1719^ No significant correlation was seen between overweight BMI and the number of oocytes retrieved (SMD=−0.07, 95% CIs −0.15 to 0.01, p=0.10) (figure 2A). Obesity was associated with a reduction in number of mature oocytes retrieved (SMD=−0.23, 95% CIs −0.41 to −0.04, p=0.01) (figure 2B), as was overweight and obesity combined (SMD=−0.11, 95% CIs −0.18 to −0.04, p=0.002) (figure 2C).

### Clinical pregnancy rate

**Figure 3:**
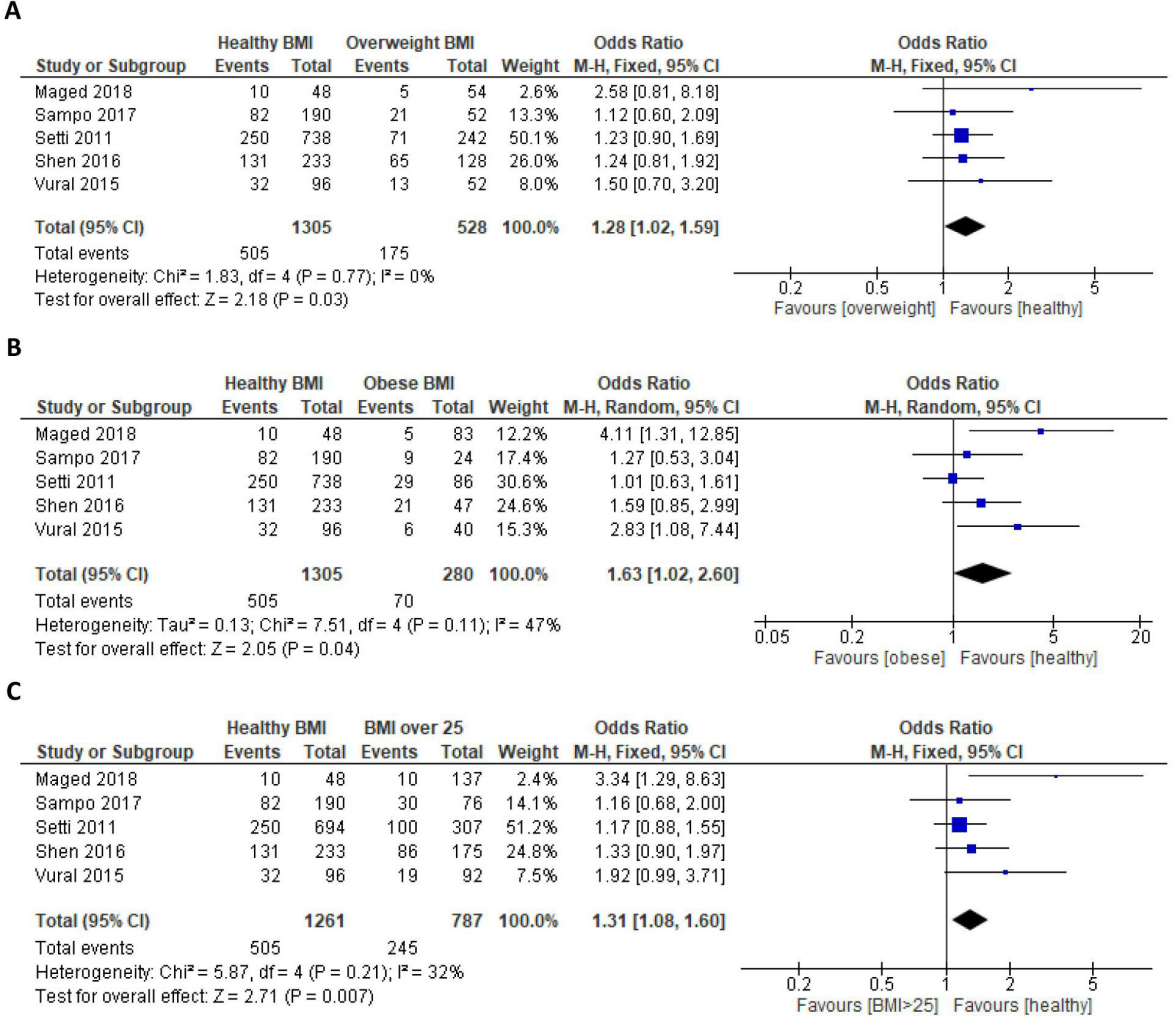
Forest plots illustrating the effects of raised body mass index (BMI) on the likelihood of clinical pregnancy. (**A**) Healthy versus overweight BMI. (B) Healthy versus obese BMI. (C) Healthy versus BMI ≥25 kg/m^2^. df, degrees of freedom; M-H, Mantel-Haenszel.

Regarding CPR, five studies^1517 20^,^22^ had data suitable for comparison in a meta-analysis. Compared with healthy weight females, those who were overweight were significantly less likely to achieve a clinical pregnancy (OR 0.78, 95% CIs 0.63 to 0.98, p=0.03) (figure 3A). Similarly, obese females had a significantly poorer chance of achieving a clinical pregnancy (OR 0.61, 95% CIs 0.39 to 0.98, p=0.04) (figure 3B); therefore, CPR was reduced in those with a BMI ≥25 (OR 0.76, 95% CIs 0.62 to 0.93, p=0.007) (figure 3C).

### Miscarriage rate

Miscarriage data were available from four studies.^15 19 21 22^ No statistical differences in miscarriage rate were identified between healthy and overweight (OR 1.16, 95% CIs 0.80 to 1.68, p=0.42) (online supplemental figure 4A), healthy and obese (OR 1.19, 95% CIs 0.71 to 1.99, p=0.52) (online supplemental figure 4B) or healthy and BMI ≥25 cohorts (OR 1.17, 95% CIs 0.84 to 1.63, p=0.35) (online supplemental figure 4C). A non-significant trend favouring healthy BMI was noted across all three cohorts (online supplemental figure 4).

### Live birth rate

Only three studies^15 18 22^ followed pregnancies until birth; therefore, three sets of LBR data were available for meta-analysis. No significant differences in LBR were identified between healthy and overweight BMI (OR 1.27, 95% CIs 0.97 to 1.67, p=0.09) (online supplemental figure 5A) or healthy and obese BMI (OR 1.20, 95% CIs 0.81 to 1.80, p=0.37) (online supplemental figure 5B), but an obvious trend towards favouring healthy weight can be seen (online supplemental figure 5A and B). A significant difference was observed between healthy BMI and BMI ≥25 (OR 1.32, 95% CIs 1.05 to 1.65, p=0.02) (online supplemental figure 5C).

## Discussion

These data, of which we are aware, are one of the first demonstrating an adverse impact of being overweight/obese on ART outcomes in women with no other diagnosed medical comorbidities and highlighted insufficient data concerning the isolated effects of BMI on natural conception.

Findings from the current study contradict previous findings that suggest overweight BMI has no significant impact on CPR,^5 7^ possibly due to excluding studies that included women with medical disorders likely to impact fertility outcomes. An association between obese BMI and reduced CPR was identified, which has previously been uncertain due to equivocal findings; Maheshwari *et al*^6^ and Ribeiro *et al*^7^ identified similar findings to the current study, contrary to Koning *et al*,^5^ who concluded that obesity does not affect CPR. While we subcategorised pregnancy outcomes to include CPR, defined as a heartbeat identified by ultrasound, a previous study^6^ aggregated all pregnancies to compare overall pregnancy rates. Although this is not directly comparable, both the current study and Maheshwari *et al*^6^ identified a reduced pregnancy rate in BMI ≥25 kg/m^2^. Increasing BMI was identified to have a more substantial effect on CPR as obesity was associated with a poorer likelihood of clinical pregnancy than overweight BMI.

A BMI ≥25.0 kg/m^2^ was associated with increased duration of ovarian stimulation, as was obese BMI compared with healthy BMI. This observation supported previous findings reported by Ribeiro *et al*.^7^

Overweight and obese BMI were deemed to have no effect on total gonadotrophin dosage; however, a trend of increasing total dose with increasing BMI was observed. Other systematic reviews^6 7^ identified both overweight and obese BMI to increase the gonadotrophin dosage requirements. However, it is possible that the large variety of gonadotrophins used in those studies included in the current meta-analysis, resulting in a high heterogeneity (I^2^ range 76–94%), prevented the identification of raised BMI impacting gonadotrophin dosage, unlike previous systematic reviews with a lesser degree of heterogeneity (I^2^ range 9.5–66.2%)^6^ (I^2^ range 54.4–64.8%).^7^

A reduced oocyte harvest was identified in those with a raised BMI (SMD=−0.11, p=0.002). Comparison of overweight BMI to healthy BMI revealed no difference in the number of mature oocytes retrieved in the ART process, whereas comparison of obese BMI to healthy BMI demonstrated a significant reduction. BMI ≥25.0 kg/m^2^ was also associated with a reduced number of oocytes retrieved. These data are akin to previous reviews identifying reduced numbers of oocytes retrieved in women with BMI ≥25.0 kg/m^2^ compared with those of a healthy weight.^6 7^

Although very few papers recorded data on miscarriages, raised BMI was not identified to affect miscarriage rate. Previous findings have reported that raised BMI significantly increases the likelihood of miscarriage.^6 7^ These contrasting findings are likely due to excluding causes of bias and the small number of studies with miscarriage data (n=4).

Most importantly, LBR was shown to be decreased in those with a BMI over 25. Previously, there has been conflicting evidence surrounding the effects raised BMI has on the LBR.^5^,^7^ A very small number of studies had LBR data (n=3), but this knowledge will aid in counselling prior to IVF when BMI is increased.

### Strengths and limitations

Strict eligibility criteria enabled exploration of the effects of increased female BMI, removing the influence of endocrine, metabolic or gynaecological disorders that have their own direct effects on BMI/fertility. By excluding females with medical comorbidities, the attainment of a relatively homogenous population was ensured, which was reflected in the heterogeneity observed in forest plots and I^2^ statistics.

Our study only examined the effect of BMI on ART outcomes, not on natural conception. One paper was identified that investigated the effects of raised BMI on natural conception,^14^ but this was excluded from our meta-analysis as the study population and measured outcomes were incomparable to all other included data sets. The study identified a significant association between increased BMI and subfecundity (a time to pregnancy of over 12 months) (OR 1.32). It is interesting to note that reducing BMI has been shown to decrease time to pregnancy.^23^ As this was the only paper identified in our search to demonstrate the effects of BMI on natural conception in women without comorbidities that affect BMI or fertility, it is apparent that further large cohort studies are required, as our meta-analysis cannot be extrapolated to natural conception.

Despite a homogenous population, these results are based on data from studies of varying quality and risk of bias. Different BMI classifications and forms of ART were used, and large differences in population sizes between the studies were noted.^15 18 22^ It is difficult to fully assess comorbidity across all included studies due to variations in study design with respect to verification of comorbidities, in addition to potentially undiagnosed comorbidities. We therefore cannot be certain that all women included in these studies had absolutely no comorbidities, which is why such a strict eligibility criterion was used to ensure as homogenous population as possible.

Outcome reporting in reproductive medicine studies is an ongoing area of debate,^24^,^27^ with the European Society for Human Reproduction and Embryology recommending ‘singleton live birth rate’ as a gold standard.^28^ Therefore, until outcomes are reported homogeneously, all pooled data should be viewed with an element of caution.^25^

Only eight studies were eligible for meta-analysis as a result of strict inclusion criteria. While this small number of studies limits the extent of findings and representativity of those receiving ART, it was necessary to adhere to strict eligibility criteria in order to ensure specific exploration of a population with no known existing endocrine, metabolic or gynaecological medical disorders, removing their potential influence on results.

### Interpretation

The current systematic review and meta-analysis demonstrate that BMI affects ART parameters and outcomes in a homogenous population, without other underlying or confounding medical comorbidities. As BMI is a modifiable risk, there is potential for improving ART outcomes by reducing excess body weight in women. These findings thus could provide useful information to impart in counselling those starting fertility treatment on the importance of a healthy BMI. Currently, in the UK, the NHS offers IVF when a female’s BMI is <30 kg/m^2^.^29^ Findings correspond with national guidance that there are increased adverse risks and poorer outcomes with IVF and increasing BMI.^29^ These data will also be helpful in an IVF setting, as an increased gonadotrophin dose and duration of stimulation, in addition to increased risks of poor IVF and pregnancy outcomes, must be considered, with women fully counselled regarding them.

### Conclusion

This review provides a unique summary of the effects of isolated increased BMI on fertility in women with no other medical comorbidities. A meta-analysis of eight ART studies demonstrated overweight and obese BMI to have significant adverse effects on ART parameters and most importantly outcomes. Our systematic review provides findings for the basis of guidance concerning ART counselling and decision-making in both public and private healthcare settings. Data surrounding the impact of isolated raised BMI in women with no other medical comorbidities on natural conception is scarce; therefore, further large primary research studies are necessary in the future in order to establish the relationship between women with no other medical comorbidities, BMI and natural conception.

## supplementary material

10.1136/bmjopen-2023-082123 online supplemental file 1

10.1136/bmjopen-2023-082123 online supplemental file 2

10.1136/bmjopen-2023-082123 online supplemental file 3

10.1136/bmjopen-2023-082123 online supplemental file 4

10.1136/bmjopen-2023-082123 online supplemental file 5

10.1136/bmjopen-2023-082123 online supplemental file 6

10.1136/bmjopen-2023-082123 online supplemental file 7

10.1136/bmjopen-2023-082123 online supplemental file 8

## Data Availability

The datasets used during the current study are all published and available from the references listed.
