# Supplementary material for: Impact of BMI on fertility in an otherwise healthy population: a systematic review and meta-analysis
Source: BMJ Open. 2024 Nov 1;14(10):e082123. doi: 10.1136/bmjopen-2023-082123 (PMC11529583; doi:10.1136/bmjopen-2023-082123)
Supplement: online supplemental file 1 [file bmjopen-14-10-s001.pdf]

Table S1. Search strategy

MEDLINE

1	(Female OR Woman OR Women)	10681742
2	(BMI OR Body mass index OR Overweight OR Obesity)	496222
3	(Infertility OR Conception OR Ovulation OR anovulation Or Fecundity).	225960
4	1 and 2 and 3	5361
5	4 and limited to English, human studies published between January 2000- July 2023	4312

Cochrane CENTRAL

1	(Female OR Woman OR Women)	2533
2	(BMI OR Body mass index OR Overweight OR Obesity)	237
3	(Infertility OR Conception OR Ovulation OR anovulation Or Fecundity).	845
4	1 and 2 and 3	22
5	4 and limited to English, human studies published between January 2000- July 2023	20

Embase

1	(Female OR Woman OR Women)	9829843
2	(BMI OR Body mass index OR Overweight OR Obesity)	589381
3	(Infertility OR Conception OR Ovulation OR anovulation Or Fecundity).	259115
4	1 and 2 and 3	6694
5	4 and limited to English, human studies published between January 2000- July 2023	5589
