# Supplementary material for: Impact of BMI on fertility in an otherwise healthy population: a systematic review and meta-analysis
Source: BMJ Open. 2024 Nov 1;14(10):e082123. doi: 10.1136/bmjopen-2023-082123 (PMC11529583; doi:10.1136/bmjopen-2023-082123)
Supplement: online supplemental file 2 [file bmjopen-14-10-s002.pdf]

Table S2. Study characteristics of the eight studies included in the meta-analysis. All studies explored the effects of raised BMI on ART outcomes.

| Study | Study design | BMI categories | | | Fertility treatment | Age (mean ± SD) | | | Number of patients/cycles | | |
|---|---|---|---|---|---|---|---|---|---|---|---|
| | | Normal | Overweight | Obese | | Normal | Overweight | Obese | Normal | Overweight | Obese |
| **Esinler et al. (2008)** | Retrospective cohort study | 18.5-24.9 | 25.0-29.9 | ≥30.0 | ICSI | 31.9 ± 4.2 | 32.5 ± 4.4 | 32.5 ± 4.5 | 627 | 339 | 147 |
| **Maged et al. (2018)** | Prospective cohort study | 18.5-24.9 | 25.0-29.9 | ≥30.0 | ICSI | 35.31 ± 5.49 | 34.70 ± 6.10 | 36.46 ± 4.90 | 48 | 54 | 83 |
| **Sampo et al. (2017)** | Retrospective cohort study | 18.5-24.9 | 25.0-29.9 | ≥30.0 | ICSI | 34.5±2.3 | 34.4±2.4 | 34.3±2.2 | 190 | 52 | 24 |
| **Setti et al. (2011)** | Retrospective cohort study | 19-24.9 | 25.0-29.9 | ≥30.0 | ICSI | 34.5 ± 4.6 | 35.2 ± 4.6 | 35.8 ± 5.1 | 738 | 242 | 86 |
| **Shen et al. (2016)** | Retrospective cohort study | 18.5-23.9 | 24-27.9 | ≥28.0 | IVF | 32.6±3.72 | 33.15±4.19 | 34.14±4.58 | 234 | 130 | 47 |
| **Shehata (2017)** | Retrospective cohort study | 18-24.9 | ≥25.0 | | ICSI | | | | 100 | 100 | |
| **Vural et al. (2015)** | Retrospective cohort study | 18.5-24.9 | 25.0-29.9 | ≥30.0 | IVF | 35.3 ± 4.8 | 34.3 ± 4.8 | 34.3 ± 4.9 | 96 | 52 | 40 |
| **Zhang et al. (2017)** | Retrospective cohort study | 18.5-24.9 | 25.0-29.9 | ≥30.0 | IVF | 32.85 ± 4.25 | 32.80 ± 4.21 | 32.85 ± 4.33 | 531 | 128 | 20 |
