# Supplementary material for: Impact of BMI on fertility in an otherwise healthy population: a systematic review and meta-analysis
Source: BMJ Open. 2024 Nov 1;14(10):e082123. doi: 10.1136/bmjopen-2023-082123 (PMC11529583; doi:10.1136/bmjopen-2023-082123)
Supplement: online supplemental file 3 [file bmjopen-14-10-s003.pdf]

Table S2. Quality and risk of bias assessment of studies included in the meta-analysis.

Study	Representativeness of exposed cohort	Selection of non-exposed cohort	Ascertainment of exposure	Demonstration that outcome of interest was not present at start of study	Comparability of cohorts on the basis of the design or analysis	Assessment of outcome	Was follow up long enough for outcome to occur	Adequacy of follow up of cohorts	Score
Esinler 2008 [27]	★	★	★	★	-	★	★	-	6
Maged 2018 [10]	★	★	★	★	-	★	★	-	6
Sampo 2017 [29]	★	★	★	★	★	★	★	★	8
Setti 2011 [30]	★	★	★	★	★	★	★	-	7
Shehata 2017 [26]	★	★	★	★	-	★	★	-	6
Shen 2016 [24]	★	★	★	★	-	★	★	-	6
Vural 2015 [28]	★	★	★	★	★	★	★	-	7
Zhang 2017 [25]	★	★	★	★	★	★	★	-	7

Thresholds for converting the Newcastle-Ottawa scales to Agency for Health Research and Quality (AHRQ) standards (good, fair and poor):

- Good quality: 3 or 4 stars in selection domain AND 1 or 2 stars in comparability domain AND 2 or 3 stars in outcome/exposure domain
- Fair quality: 2 stars in selection domain AND 1 or 2 stars in comparability domain AND 2 or 3 stars in outcome/exposure domain
- Poor quality: 0 or 1 star in selection domain OR 0 stars in comparability domain OR 0 or 1 star in outcome/exposure domain
