# Supplementary material for: Impact of BMI on fertility in an otherwise healthy population: a systematic review and meta-analysis
Source: BMJ Open. 2024 Nov 1;14(10):e082123. doi: 10.1136/bmjopen-2023-082123 (PMC11529583; doi:10.1136/bmjopen-2023-082123)

Figure S1. Visual analysis of funnel plot revealed no obvious publication bias. Blue dashed lines represent 95% confidence intervals.

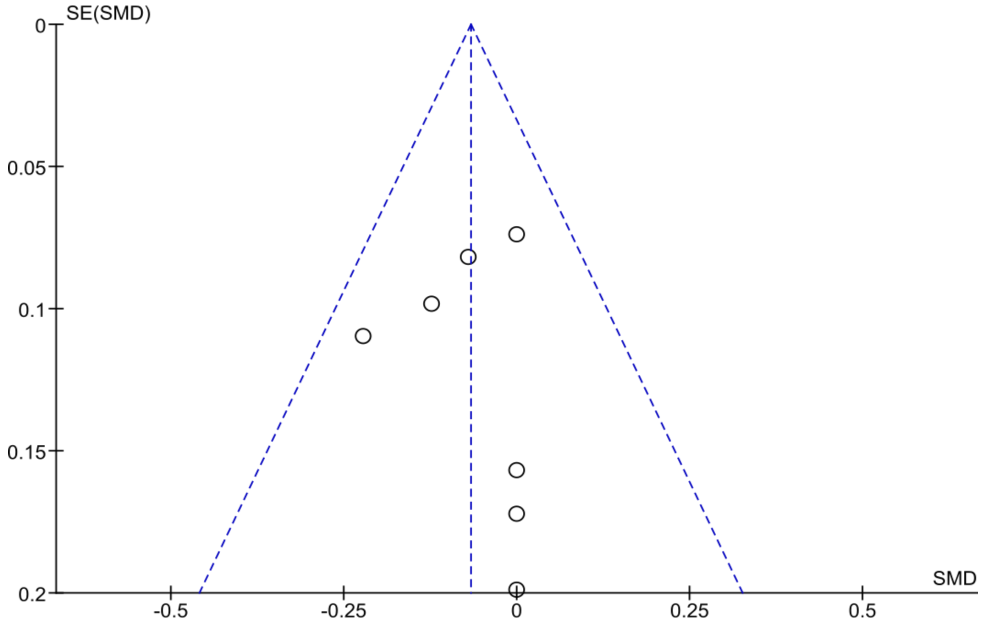

Supplement: online supplemental file 4 [file bmjopen-14-10-s004.pdf]
