# Supplementary material for: Impact of BMI on fertility in an otherwise healthy population: a systematic review and meta-analysis
Source: BMJ Open. 2024 Nov 1;14(10):e082123. doi: 10.1136/bmjopen-2023-082123 (PMC11529583; doi:10.1136/bmjopen-2023-082123)

Figure S2. Forest plot comparison of total gonadotrophin dosage in A) Healthy versus overweight BMI B) Healthy versus obese BMI and C) Healthy versus BMI ≥ 25.

A

Study or Subgroup	Healthy BMI Mean	SD	Total	Overweight BMI Mean	SD	Total	Weight	Std. Mean Difference IV, Random, 95% CI
Esinler 2008	2,516.5	1,057.2	451	2,779.1	1,160.1	222	17.2%	-0.24 [-0.40, -0.08]
Maged 2018	4,176.6	858.1	48	3,886.1	801.3	54	9.9%	0.35 [-0.04, 0.74]
Sampo 2017	2,043	489	190	1,940	536	52	12.3%	0.21 [-0.10, 0.51]
Setti 2011	2,314	666.1	738	2,322.4	676.5	242	17.7%	-0.01 [-0.16, 0.13]
Shen 2016	1,982.93	566.38	234	2,216.71	517.91	130	15.3%	-0.42 [-0.64, -0.21]
Vural 2015	2,993	1,212	96	2,772	844	52	11.4%	0.20 [-0.14, 0.54]
Zhang 2017	2,732.9	723.7	531	2,810.5	683.4	128	16.1%	-0.11 [-0.30, 0.08]
**Total (95% CI)**			**2288**			**880**	**100.0%**	**-0.04 [-0.22, 0.13]**

Heterogeneity: $Tau^2 = 0.04$; $Chi^2 = 24.77$, df = 6 (P = 0.0004); $I^2 = 76\%$
Test for overall effect: Z = 0.50 (P = 0.62)

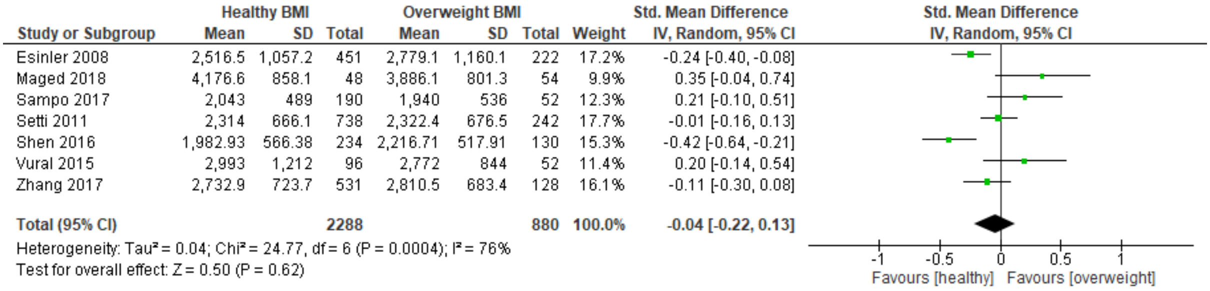

B

Study or Subgroup	Healthy BMI Mean	SD	Total	Obese BMI Mean	SD	Total	Weight	Std. Mean Difference IV, Random, 95% CI
Esinler 2008	2,516.5	1,057.2	451	3,248.3	1,255.1	102	15.5%	-0.67 [-0.89, -0.45]
Maged 2018	4,176.6	858.1	48	4,145.7	1,030.9	83	14.2%	0.03 [-0.32, 0.39]
Sampo 2017	2,043	489	190	2,109	605	24	13.4%	-0.13 [-0.56, 0.29]
Setti 2011	2,314	666.1	738	2,318.7	647.6	86	15.5%	-0.01 [-0.23, 0.22]
Shen 2016	1,982.93	566.38	234	2,636.43	673.99	47	14.5%	-1.11 [-1.44, -0.79]
Vural 2015	2,993	1,212	96	2,752.8	1,003	40	14.0%	0.21 [-0.16, 0.58]
Zhang 2017	2,732.9	723.7	531	3,195.6	883.6	20	13.1%	-0.63 [-1.08, -0.19]
**Total (95% CI)**			**2288**			**402**	**100.0%**	**-0.33 [-0.69, 0.02]**

Heterogeneity: $Tau^2 = 0.20$; $Chi^2 = 53.79$, df = 6 (P < 0.00001); $I^2 = 89\%$
Test for overall effect: Z = 1.82 (P = 0.07)

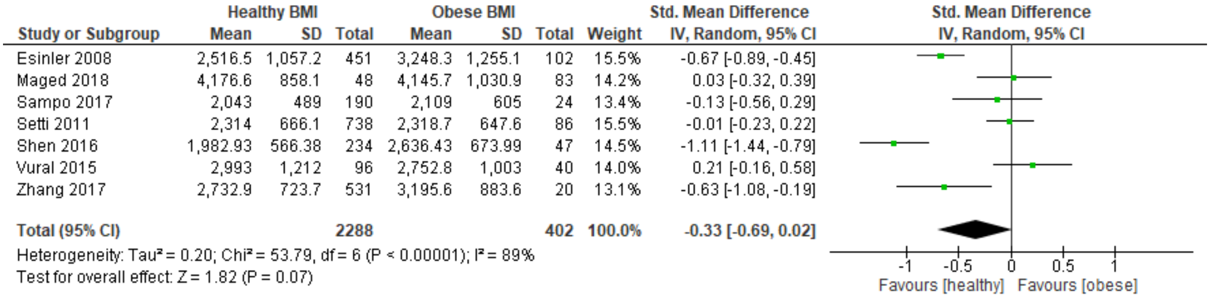

C

Study or Subgroup	Healthy BMI Mean	SD	Total	BMI over 25 Mean	SD	Total	Weight	Std. Mean Difference IV, Random, 95% CI
Esinler 2008	2,516.5	1,057.2	451	2,926.8	1,190	324	15.0%	-0.37 [-0.51, -0.22]
Maged 2018	4,176.6	858.1	48	4,043.4	940.9	137	13.1%	0.14 [-0.18, 0.47]
Sampo 2017	2,043	489	190	1,993.4	557.8	76	13.8%	0.10 [-0.17, 0.36]
Setti 2011	2,314	666.1	738	2,321.4	668.9	328	15.1%	-0.01 [-0.14, 0.12]
Shen 2016	1,982.9	566.4	234	2,328.2	559.4	177	14.5%	-0.61 [-0.81, -0.41]
Vural 2015	2,993	1,212	96	2,763.7	913.1	92	13.6%	0.21 [-0.07, 0.50]
Zhang 2017	531	2,732.9	7237	2,862.5	710.5	148	14.8%	-0.86 [-1.02, -0.70]
**Total (95% CI)**			**8994**			**1282**	**100.0%**	**-0.21 [-0.51, 0.09]**

Heterogeneity: $Tau^2 = 0.15$; $Chi^2 = 102.28$, df = 6 (P < 0.00001); $I^2 = 94\%$
Test for overall effect: Z = 1.38 (P = 0.17)

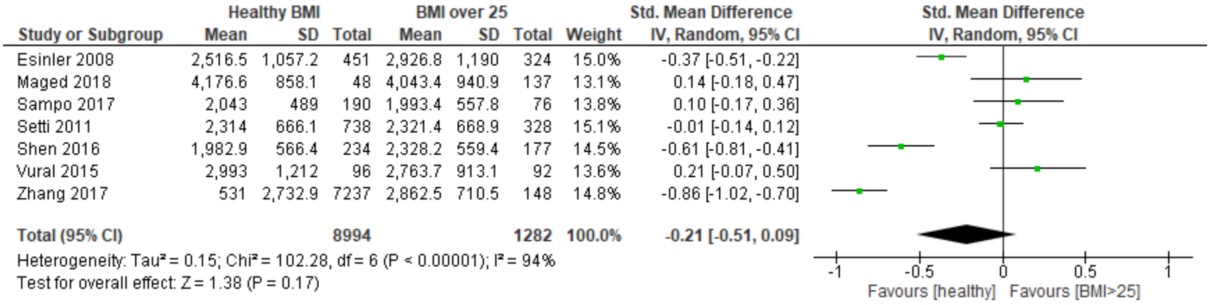

Supplement: online supplemental file 5 [file bmjopen-14-10-s005.pdf]
