# Supplementary material for: Impact of BMI on fertility in an otherwise healthy population: a systematic review and meta-analysis
Source: BMJ Open. 2024 Nov 1;14(10):e082123. doi: 10.1136/bmjopen-2023-082123 (PMC11529583; doi:10.1136/bmjopen-2023-082123)

Figure S3. Forest plot comparison of duration of ovarian stimulation in A) Healthy versus overweight BMI B) Healthy versus obese BMI and C) Healthy versus BMI ≥ 25.

A

Study or Subgroup	Healthy BMI Mean	SD	Total	Overweight BMI Mean	SD	Total	Weight	Std. Mean Difference IV, Fixed, 95% CI
Esinler 2008	10.1	1.7	451	10.3	2.7	222	34.2%	-0.10 [-0.26, 0.06]
Maged 2018	11.83	1.83	48	11.39	1.55	54	5.8%	0.26 [-0.13, 0.65]
Sampo 2017	7.5	1.4	190	7.6	1.1	52	9.4%	-0.07 [-0.38, 0.23]
Shen 2016	9.12	1.48	234	8.99	1.44	130	19.2%	0.09 [-0.13, 0.30]
Vural 2015	8.5	2.5	96	8.6	2	52	7.8%	-0.04 [-0.38, 0.29]
Zhang 2017	10.91	7.37	531	11.48	7.27	128	23.7%	-0.08 [-0.27, 0.12]
**Total (95% CI)**			**1550**			**638**	**100.0%**	**-0.03 [-0.12, 0.06]**

Heterogeneity: Chi² = 4.24, df = 5 (P = 0.52); I² = 0%
Test for overall effect: Z = 0.61 (P = 0.54)

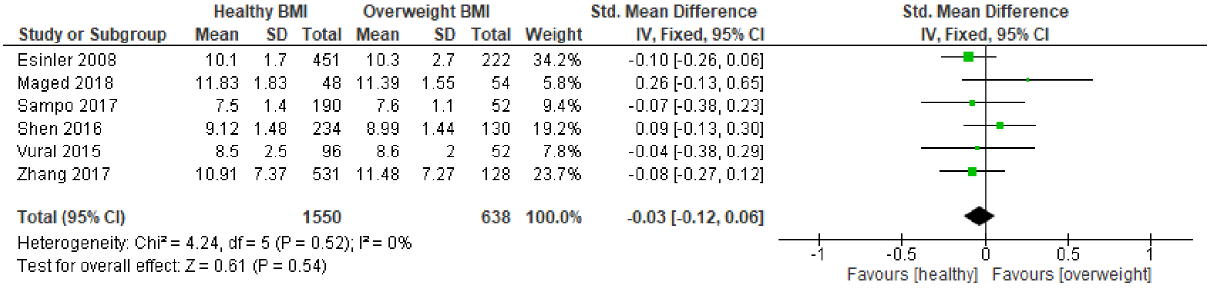

B

Study or Subgroup	Healthy BMI Mean	SD	Total	Obese BMI Mean	SD	Total	Weight	Std. Mean Difference IV, Fixed, 95% CI
Esinler 2008	10.1	1.7	451	10.6	2.6	102	37.5%	-0.26 [-0.48, -0.05]
Maged 2018	11.83	1.83	48	12.07	1.77	83	13.7%	-0.13 [-0.49, 0.22]
Sampo 2017	7.5	1.4	190	7.8	1.3	24	9.6%	-0.22 [-0.64, 0.21]
Shen 2016	9.12	1.48	234	9.44	1.38	47	17.7%	-0.22 [-0.53, 0.10]
Vural 2015	8.5	2.5	96	8.6	3.1	40	12.8%	-0.04 [-0.41, 0.33]
Zhang 2017	10.91	7.37	531	12.85	8	20	8.7%	-0.26 [-0.71, 0.18]
**Total (95% CI)**			**1550**			**316**	**100.0%**	**-0.20 [-0.34, -0.07]**

Heterogeneity: Chi² = 1.30, df = 5 (P = 0.93); I² = 0%
Test for overall effect: Z = 3.03 (P = 0.002)

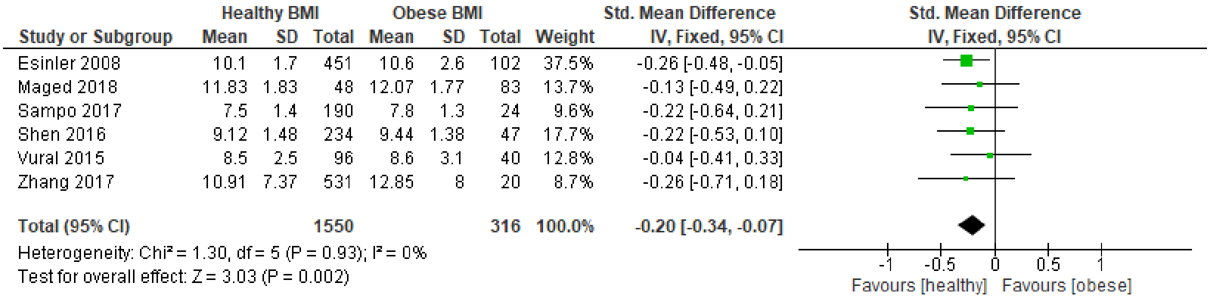

C

Study or Subgroup	Healthy BMI Mean	SD	Total	BMI over 25 Mean	SD	Total	Weight	Std. Mean Difference IV, Fixed, 95% CI
Esinler 2008	10.1	1.7	451	10.39	2.67	324	30.3%	-0.13 [-0.28, 0.01]
Maged 2018	11.83	1.83	48	11.8	1.68	137	5.7%	0.02 [-0.31, 0.35]
Sampo 2017	7.5	1.4	190	7.66	1.16	76	8.7%	-0.12 [-0.39, 0.15]
Shehta 2017	12.37	1.93	100	12.56	2.31	100	8.0%	-0.09 [-0.37, 0.19]
Shen 2016	9.12	1.48	234	9.11	1.42	177	16.2%	0.01 [-0.19, 0.20]
Vural 2015	8.5	2.5	96	8.6	2.48	92	7.6%	-0.04 [-0.33, 0.25]
Zhang 2017	10.91	7.37	7237	11.67	7.37	148	23.4%	-0.10 [-0.27, 0.06]
**Total (95% CI)**			**8356**			**1054**	**100.0%**	**-0.08 [-0.16, -0.00]**

Heterogeneity: Chi² = 1.88, df = 6 (P = 0.93); I² = 0%
Test for overall effect: Z = 2.07 (P = 0.04)

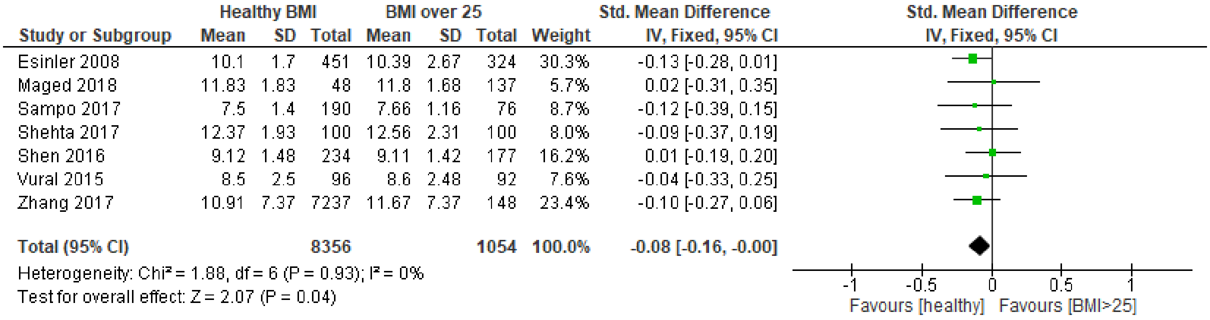

Supplement: online supplemental file 6 [file bmjopen-14-10-s006.pdf]
