# Supplementary material for: Impact of BMI on fertility in an otherwise healthy population: a systematic review and meta-analysis
Source: BMJ Open. 2024 Nov 1;14(10):e082123. doi: 10.1136/bmjopen-2023-082123 (PMC11529583; doi:10.1136/bmjopen-2023-082123)

Figure S4. Analysis of miscarriage data available from four studies. A) Normal versus overweight BMI B) Normal versus obese BMI and C) Normal versus BMI ≥ 25.

A

	Healthy BMI		Overweight BMI			Odds Ratio
**Study or Subgroup**	**Events**	**Total**	**Events**	**Total**	**Weight**	**M-H, Fixed, 95% CI**
Esinler 2008	34	251	17	123	33.2%	0.98 [0.52, 1.83]
Sampo 2017	11	82	4	21	9.3%	0.66 [0.19, 2.32]
Setti 2011	43	250	19	71	41.2%	0.57 [0.31, 1.06]
Shen 2016	25	131	9	65	16.4%	1.47 [0.64, 3.36]
**Total (95% CI)**		**714**		**280**	**100.0%**	**0.86 [0.59, 1.24]**
Total events	113		49			

Heterogeneity: Chi² = 3.65, df = 3 (P = 0.30); I² = 18%
Test for overall effect: Z = 0.80 (P = 0.42)

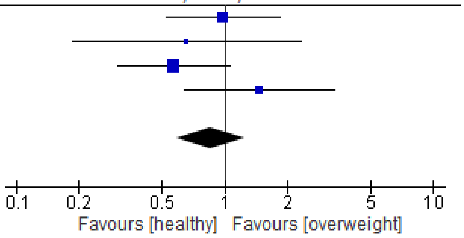

B

	Healthy BMI		Obese BMI			Odds Ratio
**Study or Subgroup**	**Events**	**Total**	**Events**	**Total**	**Weight**	**M-H, Fixed, 95% CI**
Esinler 2008	34	251	11	58	51.5%	0.67 [0.32, 1.42]
Sampo 2017	11	82	1	9	5.2%	1.24 [0.14, 10.90]
Setti 2011	43	250	5	29	24.7%	1.00 [0.36, 2.76]
Shen 2016	25	131	4	21	18.6%	1.00 [0.31, 3.24]
**Total (95% CI)**		**714**		**117**	**100.0%**	**0.84 [0.50, 1.41]**
Total events	113		21			

Heterogeneity: Chi² = 0.67, df = 3 (P = 0.88); I² = 0%
Test for overall effect: Z = 0.65 (P = 0.52)

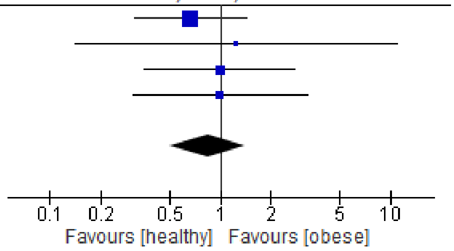

C

	Healthy BMI		BMI over 25			Odds Ratio
**Study or Subgroup**	**Events**	**Total**	**Events**	**Total**	**Weight**	**M-H, Fixed, 95% CI**
Esinler 2008	34	251	28	181	37.2%	0.86 [0.50, 1.47]
Sampo 2017	11	82	5	30	8.4%	0.77 [0.24, 2.45]
Setti 2011	43	250	24	100	37.6%	0.66 [0.37, 1.16]
Shen 2016	25	131	13	86	16.8%	1.32 [0.64, 2.76]
**Total (95% CI)**		**714**		**397**	**100.0%**	**0.85 [0.61, 1.19]**
Total events	113		70			

Heterogeneity: Chi² = 2.22, df = 3 (P = 0.53); I² = 0%
Test for overall effect: Z = 0.94 (P = 0.35)

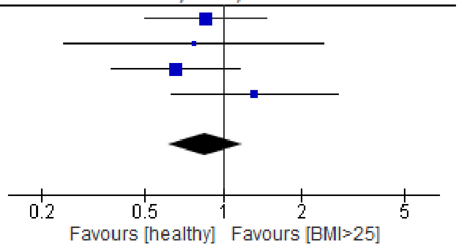

Supplement: online supplemental file 7 [file bmjopen-14-10-s007.pdf]
