# Supplementary material for: Impact of BMI on fertility in an otherwise healthy population: a systematic review and meta-analysis
Source: BMJ Open. 2024 Nov 1;14(10):e082123. doi: 10.1136/bmjopen-2023-082123 (PMC11529583; doi:10.1136/bmjopen-2023-082123)

Figure S5. Data for live births was available from three studies, one of which included overweight and obese BMI pooled together. A) Normal versus overweight BMI B) Normal versus obese BMI C) Normal versus BMI ≥ 25.

A

Study or Subgroup	Healthy BMI Events	Healthy BMI Total	Overweight BMI Events	Overweight BMI Total	Weight	Odds Ratio M-H, Fixed, 95% CI
Setti 2011	203	694	51	225	58.0%	1.41 [0.99, 2.01]
Shen 2016	106	233	56	128	42.0%	1.07 [0.70, 1.66]
**Total (95% CI)**		**927**		**353**	**100.0%**	**1.27 [0.97, 1.67]**
Total events	309		107			

Heterogeneity: Chi² = 0.92, df = 1 (P = 0.34); I² = 0%
Test for overall effect: Z = 1.71 (P = 0.09)

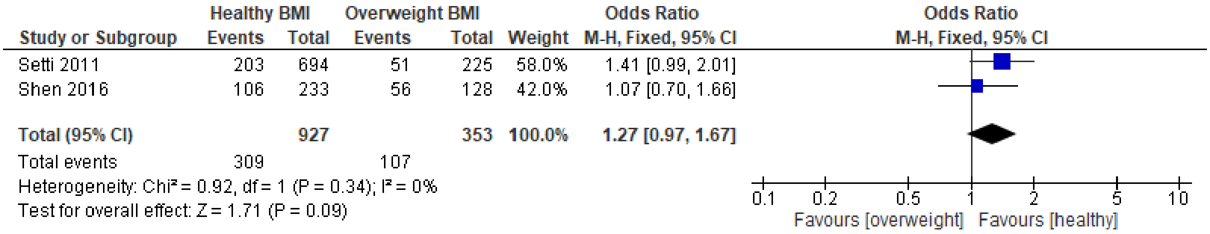

B

Study or Subgroup	Healthy BMI Events	Healthy BMI Total	Obese BMI Events	Obese BMI Total	Weight	Odds Ratio M-H, Fixed, 95% CI
Setti 2011	203	694	23	82	65.4%	1.06 [0.64, 1.76]
Shen 2016	106	233	17	47	34.6%	1.47 [0.77, 2.82]
**Total (95% CI)**		**927**		**129**	**100.0%**	**1.20 [0.81, 1.80]**
Total events	309		40			

Heterogeneity: Chi² = 0.61, df = 1 (P = 0.43); I² = 0%
Test for overall effect: Z = 0.90 (P = 0.37)

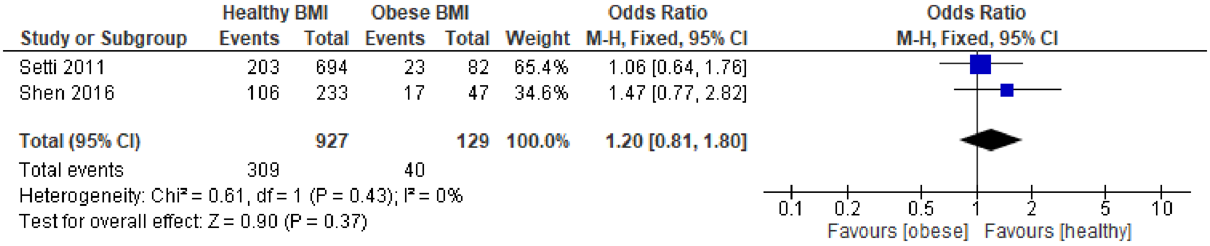

C

Study or Subgroup	Healthy BMI Events	Healthy BMI Total	BMI over 25 Events	BMI over 25 Total	Weight	Odds Ratio M-H, Fixed, 95% CI
Setti 2011	203	694	74	307	54.4%	1.30 [0.96, 1.77]
Shehata 2017	38	100	25	100	11.6%	1.84 [1.00, 3.37]
Shen 2016	106	233	73	175	34.0%	1.17 [0.78, 1.73]
**Total (95% CI)**		**1027**		**582**	**100.0%**	**1.32 [1.05, 1.65]**
Total events	347		172			

Heterogeneity: Chi² = 1.53, df = 2 (P = 0.47); I² = 0%
Test for overall effect: Z = 2.40 (P = 0.02)

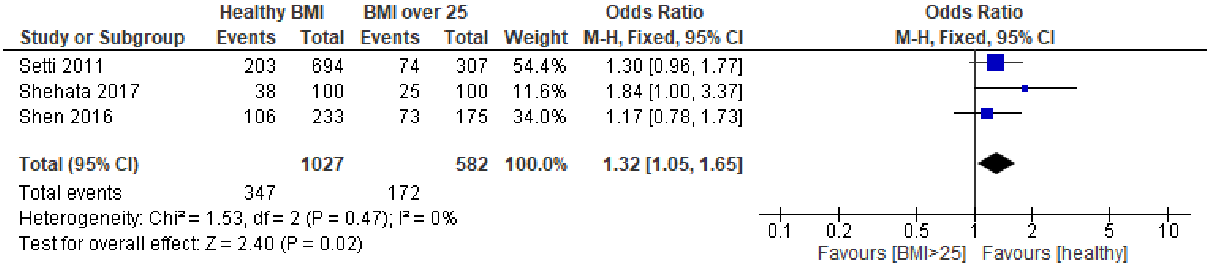

Supplement: online supplemental file 8 [file bmjopen-14-10-s008.pdf]
