# Supplementary material for: Impact of BMI on fertility in an otherwise healthy population: a systematic review and meta-analysis
Source: BMJ Open. 2024 Nov 1;14(10):e082123. doi: 10.1136/bmjopen-2023-082123 (PMC11529583; doi:10.1136/bmjopen-2023-082123)
Supplement: Supplementary file 9 [file bmjopen-2023-082123.reviewer_comments.pdf]

PEER REVIEW HISTORY

BMJ Open publishes all reviews undertaken for accepted manuscripts. Reviewers are asked to complete a checklist review form and are provided with free text boxes to elaborate on their assessment. These free text comments are reproduced below.

ARTICLE DETAILS

Title (Provisional)

The impact of BMI on fertility in an otherwise healthy population: a systematic review and meta-analysis

Authors

Turner, Florence; Powell, Simon G; Al-Lamee, Hannan; Gadhvi, Anjali; Palmer, Ellen; Drakeley, Andrew; Sprung, Victoria S; Hapangama, Dharani; Tempest, Nicola

---

VERSION 1 - REVIEW

**Reviewer**	**1**
**Name**	**Thurin, Ann**
**Affiliation**	**University of Gothenburg**
**Date**	**20-Dec-2023**
**COI**	**I haveno competing interests**

The study is a systematic review and meta-analysis on effect of BMI on fertility outcomes in female with no other co-morbidity.

There are two major problems. Although the subject per se is interesting and the search has been performed in a scientific way, unfortunately the study has not been able to investigate the issue in a convincing manner. The authors started out with many studies and then ended up with only eight included in the analysis, of which only three could be assessed for the most important outcome, live birth. Authors have excluded all studies where women were diagnosed PCOS or other co-morbidities, and this was the purpose of the study. But how can we know that the remaining studies from China, Egypt, Turkey, Argentine and Brazil had really checked co-morbidity in the women? Table one does not show exactly how the process of elimination was performed. And is it at all relevant to investigate overweight and obese women and exclude different conditions that are known to be increased in this group? There are many much larger studies showing a negative impact on live birth increasing with BMI, but unfortunately not included in references. Even if some of these women have PCOS, that might not be a problem, nor substituted hypothyroidism.

Another miscomprehension is that obesity is “modifiable”. It is widely known that it is indeed very difficult to lose weight and even more difficult to maintain the lower weight

achieved for a longer period. Thus, it is very questionable that obesity really is modifiable. The four RCTs studying weight reduction before fertility treatment have shown no improved live birth rates. Authors have not included relevant RCTs in the references.

**Reviewer**	**2**
**Name**	**Proietto, J**
**Affiliation**	**university of Melbourne, Medicine**
**Date**	**02-Apr-2024**
**COI**	**None**

This paper is a review and metanalysis of studies investigating the impact of obesity on fertility. The unique issue of this review is that they only chose studies that excluded women that may have had other causes of infertility apart from obesity confirming that both overweight and obesity reduce fertility. The women in the studies chosen had excluded women with endocrine, metabolic or Gynaecological disorders. One other line of evidence that supports the conclusion of this study is the fact that weight loss reduces time to conception when there was no need to treat any other factors that could interfere with conception. (Price S et al. Time to pregnancy after a prepregnancy very-low-energy diet program in women with obesity: substudy of a randomized controlled trial. Fertil. Steril 114:1256-1262 2020).

Minor points

Line 81 The cost of obesity being 9.7 billion Pounds. Is this per year or per 10 years. Please state the duration.

**Reviewer**	**3**
**Name**	**Martin, James**
**Affiliation**	**University of Birmingham, Institute of Applied Health Research**
**Date**	**14-Jun-2024**
**COI**	**No competing interests**

This manuscript is a systematic review to understand the relationship between BMI and fertility outcomes – in females with no other health issues. It addresses a gap in the literature since most other research papers don't consider the effects of other confounding

health conditions. However, most of the included studies relate to assisted reproductive technology – so the results may not be generalisable to natural conception.

In the abstract, I think it would be better to present the confidence intervals rather than the p-values, so that a reader can ascertain the likely magnitude of effect better.

The exclusion criteria is studies that included women with any comorbidities. I would like a little more information on how this was identified. Was this based on inclusion criteria within the studies, the characteristics of women within these studies or something else?

Its not clear, but I am assuming that studies of any design were included. Or at least there were no limits in the inclusion/exclusion criteria related to this.

Within the statistical analysis, you seem to imply that the I2 statistic is a test for between-study heterogeneity, which determines whether a fixed or random effects model should be used. Please note that the I2 simply measures the total variability due to between-study heterogeneity.

The studies were from various countries. It would be good to see some thoughts on whether there are differences in the processes of IVF or ICSI between countries, and differences in patient populations who are undergoing them (e.g. age, demographics, ethnicity), since the study characteristics presented in Table 2 are limited.

I think the PRISMA flow diagram is missing an arrow from screening to included. I think there should be an arrow from "Reports screened by full text (n=165)" to "Studies included in the review (n=9)"

For Figure 2 A. I think you may have the headings of "overweight BMI" and "healthy BMI" the wrong way round. As it doesn't match to the healthy BMI in parts B and C, and would also mean the mean difference was the opposite way around.

On Figure S2 and S3 and S4, you present healthy BMI on the right hand side, whereas for earlier Figures, you had presented healthy first. I think it would help to be consistent across the figures.

For Figure S5, there looks to be some labelling errors. In S5A, the Setti study has 106/738 in healthy BMI, whereas in S5B, it has 203/234 (which were the values for Shen in S5A).

VERSION 1 - AUTHOR RESPONSE

n/a

Reviewer: 1
Dr. Ann Thurin, University of Gothenburg
Comments to the Author:
The study is a systematic review and meta-analysis on effect of BMI on fertility outcomes in female with no other co-morbidity.

There are two major problems. Although the subject per se is interesting and the search has been performed in a scientific way, unfortunately the study has not been able to investigate the issue in a convincing manner. The authors started out with many studies and then ended up with only eight included in the analysis, of which only three could be assessed for the most important outcome, live birth. Authors have excluded all studies where women were diagnosed PCOS or other co-morbidities, and this was the purpose of the study. But how can we know that the remaining studies from China, Egypt, Turkey, Argentine and Brazil had really checked co-morbidity in the women? Table one does not show exactly how the process of elimination was performed. And is it at all relevant to investigate overweight and obese women and exclude different conditions that are known to be increased in this group? There are many much larger studies showing a negative impact on live birth increasing with BMI, but unfortunately not included in references. Even if some of these women have PCOS, that might not be a problem, nor substituted hypothyroidism.
Another miscomprehension is that obesity is "modifiable". It is widely known that it is indeed very difficult to lose weight and even more difficult to maintain the lower weight achieved for a longer period. Thus, it is very questionable that obesity really is modifiable. The four RCTs studying weight reduction before fertility treatment have shown no improved live birth rates. Authors have not included relevant RCTs in the references.

We thank the reviewer for highlighting their concerns. We have presented the data available with regards to BMI and fertility in a population not known to have any other co-morbidities related to fertility. Live birth is always the outcome that we would hope to present on, as this is what matters to the patients that we see and treat, but unfortunately numerous manuscripts that are published still do not follow up to live birth. We do highlight that this is a limitation and also comment that outcome reporting in reproductive medicine studies is an ongoing area of debate with the European Society for Human Reproduction and Embryology (ESHRE) recommending 'singleton live birth rate' as gold standard.

We understand that conditions such as PCOS affect women that are overweight but we also know that not all overweight women have conditions such as PCOS. PCOS comes with lots of sequelae that can affect fertility, making it more difficult for women to conceive and lose weight, therefore, we felt it was important to attempt to investigate a homogenous population that had not received any pathological diagnosis with regards to their fertility.

We agree that weight loss is very difficult and maintaining the weight loss long-term, after initial weight loss, to be even more difficult. However, considering many other absolutely unmodifiable risks such as tubal factor or maternal age we would argue weight is modifiable. NHS England has identified weight loss as an important modifiable risk and a weight loss is a major preventative strategy alongside reducing other such risk, e.g. smoking (www.england.nhs.uk/ourwork/prevention/secondary-prevention/modifiable-risk-factors-high-impact-interventions/). Long term sequelae for mothers that are overweight and children born to mothers that are overweight are known (increased risk of obstetric and neonatal complications such as gestational diabetes, hypertensive disorders of pregnancy, caesarean delivery, stillbirth, large of gestational age, congenital anomalies and neonatal intensive care admission) (ASRM Practice Committee 2021). Many women are unaware of these risks associated with excessive weight and also unaware of the multiple benefits of weight loss. Such information could potentially motivate women, in the prime of their lives, to adapt lifestyle changes and achieve weight loss. The RCTs studying weight reduction prior to fertility treatment showed no improvement of outcomes for IVF cycles but an increase in spontaneous pregnancy (Einarsson et al 2017). Although all studies had limitations, for example including women with a BMI<35, having a short duration of intervention, they still concluded that weight loss is in general a good plan for the obese/overweight women. In our manuscript, we want to present this data allowing focus

on weight management that may improve fertility outcomes while improving the quality of life and wellbeing of women in general.

Reviewer: 2
Dr. J Proietto, university of Melbourne
Comments to the Author:
This paper is a review and metanalysis of studies investigating the impact of obesity on fertility. The unique issue of this review is that they only chose studies that excluded women that may have had other causes of infertility apart from obesity confirming that both overweight and obesity reduce fertility. The women in the studies chosen had excluded women with endocrine, metabolic or Gynaecological disorders. One other line of evidence that supports the conclusion of this study is the fact that weight loss reduces time to conception when there was no need to treat any other factors that could interfere with conception. (Price S et al. Time to pregnancy after a prepregnancy very-low-energy diet program in women with obesity: substudy of a randomized controlled trial. Fertil. Steril 114:1256-1262 2020).

We thank the reviewer for their kind words in relation to our manuscript. The appropriate reference has been added to strengthen our argument and the need to understand more regarding BMI and natural conception.

Minor points

Line 81 The cost of obesity being 9.7 billion Pounds. Is this per year or per 10 years. Please state the duration.

We apologise that this was not apparent in our manuscript and we have updated this accordingly (Page 4 Line 83).

Reviewer: 3
Mr. James Martin, University of Birmingham
Comments to the Author:
This manuscript is a systematic review to understand the relationship between BMI and fertility outcomes – in females with no other health issues. It addresses a gap in the literature since most other research papers don't consider the effects of other confounding health conditions. However, most of the included studies relate to assisted reproductive technology – so the results may not be generalisable to natural conception.
In the abstract, I think it would be better to present the confidence intervals rather than the p-values, so that a reader can ascertain the likely magnitude of effect better.

Thank you for this suggestion, we have now updated the abstract appropriately with confidence intervals.

The exclusion criteria is studies that included women with any comorbidities. I would like a little more information on how this was identified. Was this based on inclusion criteria within the studies, the characteristics of women within these studies or something else?

We thank the reviewer for highlighting the lack of information for the reader to understand our inclusion criteria, we have now added more information, ensuring transparency (Page 6 Lines 123-32).

All original randomised and non-randomised studies assessing BMI in association with any pregnancy outcomes were included in this review. Studies were excluded if: (i) they did not report on BMI or any associated fertility outcome, (ii) they included any participants with comorbidities which may affect fertility such as PCOS, thyroid disease (we read all papers inclusion and exclusion criteria and if women were included that had any co-morbidities that may affect fertility we excluded the paper), (iii) were not written in the English language, (iv) were not full text articles (including abstracts and incomplete datasets), and (v) were not original research studies (including review articles, meta-analyses, case reports and conference abstracts).

Its not clear, but I am assuming that studies of any design were included. Or at least there were no limits in the inclusion/exclusion criteria related to this.

We have added further information in the study selection and eligibility criteria to make this point much clearer, thank you for alerting us to this (Page 6 Lines 123-32).

Within the statistical analysis, you seem to imply that the I2 statistic is a test for between-study heterogeneity, which determines whether a fixed or random effects model should be used. Please note that the I2 simply measures the total variability due to between-study heterogeneity.

We have now changed the wording to reflect the purpose of the I2 (Page 7 Line 156-8).

The studies were from various countries. It would be good to see some thoughts on whether there are differences in the processes of IVF or ICSI between countries, and differences in patient populations who are undergoing them (e.g. age, demographics, ethnicity), since the study characteristics presented in Table 2 are limited.

We have added more information regarding the demographics to highlight the different countries that were incorporated. The method of fertility treatment IVF/ICSI is highlighted in what is now Table 1 and age has been added where available. Ethnicity data was unfortunately not available.

I think the PRISMA flow diagram is missing an arrow from screening to included. I think there should be an arrow from “Reports screened by full text (n=165)” to “Studies included in the review (n=9)”

Thank you for highlighting this error, we have now rectified this within the PRISMA flow diagram (Figure 1).

For Figure 2 A. I think you may have the headings of “overweight BMI” and “healthy BMI” the wrong way round. As it doesn’t match to the healthy BMI in parts B and C, and would also mean the mean difference was the opposite way around.

Thank you again for highlighting this, the headings have been altered accordingly (Figure 2A).

On Figure S2 and S3 and S4, you present healthy BMI on the right hand side, whereas for earlier Figures, you had presented healthy first. I think it would help to be consistent across the figures.

We agree that we should be consistent therefore we have now altered Supplementary figures 2, 3 and 4 so that the healthy BMI title is on the left hand side.

For Figure S5, there looks to be some labelling errors. In S5A, the Setti study has 106/738 in healthy BMI, whereas in S5B, it has 203/234 (which were the values for Shen in S5A).

Thank you for pointing to this error, we have now rectified this to ensure the numbers are correct (Supplementary Figure S5B).

VERSION 2 - REVIEW

**Reviewer**	**3**
**Name**	**Martin, James**
**Affiliation**	**University of Birmingham, Institute of Applied Health Research**
**Date**	**14-Aug-2024**
**COI**	**None**

Many thanks for responding to the previous comments. I think the manuscript is much clearer as a result.

I have a couple of minor comments:

In the abstract, you use the acronym LBR without introducing it first.

Please give full details on the meta-analysis models you used – for example are you using fixed or random effects, and also consider prediction intervals and tau2. You may want to think about whether high heterogeneity in associations would be expected beforehand. In which case a random-effects model would be indicated regardless of observed heterogeneity.

VERSION 2 - AUTHOR RESPONSE

Reviewer: 3

Dr. James Martin, University of Birmingham

Comments to the Author:

Many thanks for responding to the previous comments. I think the manuscript is much clearer as a result.

We thank the reviewer for their kind words regarding the updated manuscript.

I have a couple of minor comments:

In the abstract, you use the acronym LBR without introducing it first.

Thank you for highlighting this, we have now introduced the acronym prior to its use.

Please give full details on the meta-analysis models you used – for example are you using fixed or random effects, and also consider prediction intervals and tau2. You may want to think about whether high heterogeneity in associations would be expected beforehand. In which case a random-effects model would be indicated regardless of observed heterogeneity.

Thank you for this comment, we have now ensured that the models used in the meta-analysis are clearly indicated in the manuscript (page 7 line 151-152).

VERSION 3 - REVIEW

**Reviewer**	**3**
**Name**	**Martin, James**
**Affiliation**	**University of Birmingham, Institute of Applied Health Research**
**Date**	**13-Sep-2024**
**COI**	

Thank you for addressing all the comments that had been made in the previous iterations.
